# Report of Surgical Cases Treated at the City Hospital during the Spring Term

**Published:** 1860-07

**Authors:** Geo. K. Amerman

**Affiliations:** Attending Surgeon


THE CHICAGO
MEDICAL JOURNAL.

VOL. III. July, 1860. NO. 7.

### Original Communications.

### ARTICLE 1.

### REPORT OF SURGICAL CASES

TREATED AT THE CITY HOSPITAL DURING THE SPRING TERM, BY GEO. K. AMERMAN, M. D., ATTENDING SURGEON.

CASE 1.—*Fatty Tumor—Removal—Severe Erysipelas following the Operation—Recovery.*

Mrs. J. M., aged forty, born in New York; Grandmother died of cancer of the eye; mother died at the age of fifty-four, of tetanus, the result of an injury. In early life she had an affection of the lower lip, said to be cancerous, which was removed, but returned again before the wound had entirely healed, thus rendering a second operation necessary, which had proved a permanent cure. Father died at the age of forty, from some affection of the bowels.

Mrs. M. was married at the age of twenty-seven, up to which time she enjoyed ordinary good health, having never been of a robust constitution. She has given birth to five children, three of whom are now living, the youngest aged three and a half years—all healthy. Eight years ago, whilst nursing a child, she first observed a swelling, about the size of a small egg, situated just within the scapulo-clavicular articulation. She attributed its production to the carrying the child so much on that side. The swelling, when first noticed, was free from pain and tenderness, movable, and natural in temperature. The skin covering it was normal in appearance. It felt, as she expressed it, like a piece of soft sponge. From that time till the present it has slowly increased in size, otherwise retaining its original characteristics. About two years ago she began to feel shooting pains down the arm, fore-arm, and hand. During the last six months its growth has been much more rapid than formerly. It has now attained the size of a large orange, is situated over the anterior surface of the shoulder, just below the clavicle, extending over the side of the chest, and overhanging the axilla. It is nearly globular in shape, is freely movable, having no neck, and has an elastic, almost fluid feel. The skin covering it presents a natural appearance, except, perhaps, an unusual number of veins ramifying over its surface. It is free from pain and tenderness, except when handled a length of time, which gives rise to a shooting pain down the arm. On careful examination it is found to be of different density in spots. At points there are slightly elevated nodules which are quite hard and dense, imparting the sensation of a solid floating in in some fluid. These solid substances were first observed by the patient about six months ago, being more sensitive than other parts of the tumor. The lymphatic glands in the axilla and vicinity are healthy. The general appearance of the patient is good, but not that of robust health. The local character of this tumor, its slow growth, the absence of pain, its being movable, its not having involved the surrounding parts, or neighboring lymphatics, induced me to regard it as non-malignant and recommend its removal.

The operation was performed April 4th, 1860. Two incisions extending obliquely downward and inward, from a point about half an inch from the scapular end of the clavicle, five inches in length, enclosing an elliptical portion of the integument, and extending through the skin, brought the mass fully into view, and rendered its enucleation simple and easy, by the fingers alone. One vessel, only, required the ligature. Simple dressings were used, and an anodyne ($\frac{1}{2}$ gr.

Morphia) given to quiet the patient. The anesthetic employed was Ether Sulph., which, though it produced complete anesthesia, had a very unpleasant effect after the operation, retarding reaction, and causing continued retchings and nausea for twenty-four hours. The tumor consisted of one mass of fat, thinly enveloped by a cyst wall but having numerous prolongations sent out into the surrounding parts. The whole mass was lobular, its central portion containing a solid material about the size of a small egg, of much firmer growth and consistence.

April 5.—Patient did not rest any last night, the nausea and frequent attempts to vomit continuing to harass her constantly. Pulse 120; skin hot; considerable thirst; wound glazed over throughout most of its extent and transudes a thin, bloody, serous fluid. Ordered cold water dressings to be applied, cooling draughts, and small doses of opium to allay the irritability of the stomach. At noon the excitement and febrile reaction seemed altogether disproportionate to the severity of the operation, and I was at a loss to account for it. Ordered Tr. Aconite ʒ i, tr. Opii. Camph., Spts. Nit. Dulc. āā ℥ i. Teaspoonful every three hours.

Evening—Pulse 130; skin very hot; face flushed, and pain in every part of the body. In addition to the above gave an anodyne, (1 gr. Morphia.)

April 6.—Slept some last night; nausea almost gone. Fever still high; pain in the head, back, and limbs, as yesterday; pulse 136; wound perfectly dry, the edges in apposition, but not adherent or disposed to action. All around, and extending over that side of the chest, neck, and shoulder, there is a bright red flush, indicative of the trouble and constitutional disturbance. Discontinued the cold water dressings, removed a part of the sutures, and painted the wound with the Tr. Iodine. In place of the fever mixture gave pills of Quin. et Ferri. At noon the redness had extended and the fever increased. Tr. Iodine to the wound, and in addition to the pills, the Tr. Ferri. Mur. every hour. In the evening the remainder of the sutures were removed, a poultice of flax seed applied, and 1 gr. Morphia given to procure rest.

April 7.—Slept some the latter part of last night, and feels a little more comfortable this morning. Fever slightly diminished; pulse 120, feeble; tongue coated; bowels costive. The wound discharges a small quantity of unhealthy, offensive pus and blood. Erysipelas stationary. Continued the same treatment as yesterday, with beef essence every hour. At noon more restless, and fever as severe as ever; gave a full dose of opium.

April 9.—Condition much improved since last note. Erysipelas much less; the wound discharging freely, good, healthy pus; fever almost gone. Discontinued the Tr. Ferri. Mur.; other treatment same.

April 12.—Erysipelas entirely gone; wound granulating finely; no fever; patient weak and prostrated. Continued the Quin. and Iron, and simple cerate to the wound, till it was entirely healed, which rapidly followed the subsidence of the erysipelas.

*Remarks.*—An interesting feature connected with the above case, is its hereditary history. The grandmother and mother of this patient were both affected with cancer. The first, with, probably, encephaloid of the eye, which caused her death; this being, according to some, the most active and malignant form of cancer. The latter with epithelial cancer of the lip, which is, according to many authors, the mildest form of the disease, the least malignant, and least likely to return after removal. With this hereditary predisposition, we might have anticipated cancer in this patient, and such, indeed, was the opinion of some of the surgeons who had been consulted regarding it. The hereditary tendency of cancer is a well recognized fact, but whether the children of parents affected with simple, non-malignant growths, are more liable to suffer from malignant disease than the offspring of healthy parents; or, whether the children of parents affected with cancer are more liable to suffer from simple, non-malignant growths, are questions which, as yet, remain unanswered. Paget, in his article on tumors, alludes to this subject.

Another interesting feature in the above case, was the presence of those indurated knots in its substance. These were found, on microscopical examination, to be nothing more than an interlacement of fine fibres, similar, in all respects, to the fibro-cellular tissue in other parts of the tumor. A hard mass, developed in the substance of a fatty tumor, Paget regards as the result of a slow form of inflammation, producing contraction, and hardening of the fibro-cellular tissue of the part, and he mentions a specimen contained in the Museum of St. Bartholomew's Hospital, of a fatty tumor, the seat of osseous formations. Without a knowledge of these facts, the diagnosis might be obscure and difficult. Fatty tumors yield to no treatment except extirpation by the knife, and being generally enclosed in a thin cyst, the operation is exceedingly simple and easy. The wound seldom unites by the first intention.

CASE II.—*Talipes Varus—Both Feet—Division of the Tendo-Achillis—Result Successful.*

Walter McPherson aged one year and nine months, applied as an out patient at the Hospital, March 6th, 1860, for a congenital deformity of the feet. Both feet turned inward, the soles facing each other. In walking, the weight of the body rested entirely on the outside of the feet, at which point, just over the tarso-metatarsal articulation, an artificial bursa was developed. The elevation of the heel was not a very prominent feature in this case, but any attempt to bring the feet to their normal position, caused the Tendo-Achillis to become tense and prominent. The deformity was greatest in the left foot. The treatment consisted in division of the Tendo-Achillis, the application of a sheet iron shoe with side splints extending up the leg, and the subsequent use of an ordinary shoe with a stiff sole. The operation was performed March 6th, anesthesia produced by Ether Sulph., which acted very kindly and without any unpleasant after effect. The iron shoes were immediately applied, the feet brought very nearly into proper position, and their use continued for four weeks, when they were replaced by common shoes with stiffened soles.

*Remarks.*—Tenotomy, or the sub-cutaneous division of tendons for the relief of deformities, and especially those of the feet, has now become such an established principle in the treatment of these difficulties as to be no longer a question for discussion. It, conjoined with proper mechanical means, seldom fails to entirely remedy the deformity, and when properly executed, is free from danger. Mechanical appliances form an important and essential part of the treatment, their object being to reduce the deformity and retain the member in its proper position. These, then, form the elements of treatment for club-foot. First, the division of the tendon or tendons at fault; Second, the use of such an apparatus as will reduce and prevent the recurrence of the deformity.

The division of the tendons is a simple, easy, and safe operation. The particular tendon or tendons at fault will depend on the variety of the deformity, there being four for each foot. Immediately after the division of the tendons, an apparatus must be applied of such make as to bring the foot nearly or quite into its proper position. Generally it requires some time, perhaps a week, to entirely reduce the deformity, but by perseverance it can always be accomplished, providing the seat of the difficulty was in the divided tendon. Great care is necessary during this period of the treatment, as the result will depend, almost entirely upon a proper adaptation of the mechanical appliances used. These must be carefully padded, worn day and night, and readjusted as often as they become at all loose or deranged. The length of time necessary to effect a cure varies in different cases, from four weeks to as many months, so that it is impossible to fix any definite time required to cure any particular case. The causes of this deformity, the appearances on dissection of the deformed part, the age most favorable for the treatment, and the different modifications of apparatus used, are all matters of interest and importance connected with this subject, and demand the careful attention of those who attempt their management.

Case III.—*Fracture of both Bones of the Leg—Imperfect Union—Application of Side Splints—Recovery.*

Hugh Jones, aged twenty-seven, born in Wales; habits moderately temperate; general health good; whilst engaged in blasting a tree, January 12th, 1860, a portion of its root struck him with great violence, about the middle of the left leg, producing an oblique fracture of the tibia and fibula. The soft parts were uninjured to any great extent. A surgeon was called, who immediately reduced the fracture and dressed it with compresses, two side splints, and a roller bandage. He was confined to his bed, and everything progressed favorably for three weeks without any change in the dressings or re-adjustment. At the end of this time the dressings were removed for the first, but union not being firm, they were again applied in the same way, and the part maintained at perfect rest for four weeks longer. At this period, seven weeks from the receipt of the injury, all the dressings were removed, and the patient directed to use his limb as much as possible.

March 27th, ten weeks from the date of the fracture, and three after all treatment had been discontinued, he was admitted into the City Hospital. The limb, at the time of his admission, was greatly swollen, and any attempt to use it produced severe pain at the seat of the fracture. The foot of the affected side was rotated inward, the limb having an outward curvature of considerable angularity at point of fracture. On taking hold of the limb and using gentle pressure, it readily yielded at the seat of the fracture, and could be brought almost into its proper position, the union being too imperfect to support its own weight while extended. It was dressed with a roller bandage from the toes to the knee, two well-padded side splints, and a second roller, applied rather tightly over the whole; also enjoined perfect rest. This treatment was continued for four weeks, the dressings re-adjusted weekly. At the end of that time the limb was perfectly straight and the union much more firm. A starch bandage was now applied, with pasteboard splints, and the patient allowed to go about on crutches. This was continued for three weeks longer, when all dressings were removed and the patient discharged cured, having been under treatment at the Hospital seven weeks.

*Remarks.*—The important practical feature connected with the above case is the admonition furnished us, in observing the effect produced by the too early removal of the dressings and the attempt to use the limb before union was firmly established. At the end of seven weeks everything looked favorable, the limb having united without deformity, and no doubt with considerable firmness, and had the dressings been continued a few weeks longer, the result would have been, in all probability, perfectly satisfactory. Three weeks later, and the limb is seriously deformed, movable at the seat of fracture, unable to support its own weight, swollen and painful, and this result, plainly attributable to the too early discontinuance of proper treatment. The length of time required for a perfect union in fractures of the leg, the first intimations of trouble if the dressings have been removed too early, the various plans of remedying the deformities which occasionally follow these accidents, and also the success we may anticipate in their proper management, are questions full of interest and importance, and having received, of late, careful attention and study from those eminently qualified, should be familiar to every one undertaking their management.

We have had another case somewhat similar to the above under treatment as an out-patient at the Hospital, and in this connection will give a brief abstract of the history.

Case IV.—E. L., aged nine months. About two months ago her mother observed that she favored her right arm, and flinched whenever she was carelessly handled. She, however, thought nothing serious of it, and as there was no swelling, no attention was paid to it. About three weeks ago she observed for the first, that the fore-arm was considerably bent, the convexity being on the dorsal surface. This alarmed her, and she applied as an out-patient at the City Hospital, April 17th, 1860. At the time of her application an angular curvature existed at about one inch below the elbow joint, having its convexity on the dorsal surface. The motions of the fore-arm were unimpaired; no pain on moving the part. The nature of the case was very evidently fracture of both bones, and union with deformity. The treatment consisted in the application of a dorsal and palmer splint, confined rather closely by means of a roller bandage. Dressings readjusted weekly. Discharged cured May 15th, having been under treatment nearly four weeks. One common feature in both the foregoing cases, was the presence of what has been formerly regarded as the provisional callus. In the first case, a hard, elevated ring, completely encircled both bones at the seat of the fracture, and was apparently thickest on their convexity. In the latter case, the same condition existed at the fractured part, but not so distinctly marked. In both, this callus underwent partial absorption during the treatment, and doubtless will, in time, entirely disappear. This "provisional," or "ensheathing" callus, as it is termed by Paget, he regards as uncommon in the human subject, except the part be frequently moved, and the apposition imperfectly maintained. If this reasoning is correct, we must attribute its production, in the above cases, to the improper use of the part, and must expect its occurrence only in those instances where inadequate means are employed to maintain perfect rest and apposition of the fractured bones.

(To be continued.)

